# Assessing the Accuracy of Linear Alveolar Bone Measurements for Implant Planning Using Cone-Beam Computed Tomography by Comparing Three Competent Three-Dimensional Imaging Software: An In Vitro Study

**DOI:** 10.7759/cureus.73172

**Published:** 2024-11-06

**Authors:** Ananya Neralla, Silpiranjan Mishra, Sangamesh NC, Bhuvaneshwari Srinivasan, Atul Anand Bajoria, Dhirendra Singh

**Affiliations:** 1 Oral Medicine and Radiology, Kalinga Institute of Dental Sciences, Bhubaneswar, IND; 2 Periodontology, Kalinga Institute of Dental Sciences, Bhubaneswar, IND

**Keywords:** 3d imaging software, alveolar bone, cbct, dimensional accuracy, in-vitro, linear correlation

## Abstract

Objective: This study aims to assess the accuracy of linear alveolar bone measurements for implant planning using cone-beam computed tomography (CBCT) by comparing three 3D imaging software: CareStream (Carestream Health, Rochester, New York, United States), RadiAnt (Medixant, Poznan, Poland), and iRYS (Cefla s.c., Imola, Italy).

Methods: Twenty-seven dry goat mandibles were used for this in-vitro study. Gutta-percha markers were placed on the mandibles, which were then scanned using a CBCT machine. Linear measurements of the alveolar bone were taken at the implant sites using CareStream, RadiAnt, and iRYS software, and compared to gold-standard measurements obtained using digital Vernier calipers. Measurements included bone height and width, and the absolute measurement errors were calculated to assess accuracy.

Results: iRYS consistently provided the most accurate measurements compared to the control, especially at lower error thresholds. RadiAnt tended to overestimate, while CareStream yielded intermediate values. Across all thresholds, iRYS showed the least deviation, followed by CareStream and RadiAnt. Statistical analysis showed no significant differences between the three software programs at higher error thresholds, confirming the reliability of each for implant planning.

Conclusion: All three software programs (iRYS, CareStream, and RadiAnt) offered reliable and accurate measurements for implant planning using CBCT, with iRYS being the most accurate. Clinicians can confidently use any of the three, although iRYS may be preferred for cases requiring higher precision.

## Introduction

Implant dentistry has gained prominence as a reliable method for replacing missing teeth, requiring meticulous preoperative planning to achieve successful functional and aesthetic outcomes. One of the most critical steps in this planning process is the evaluation of the alveolar bone's linear dimensions, which include both bone height and width. These parameters are essential for determining the available bone volume, selecting the appropriate implant size, and ensuring long-term implant stability [1]. Precise imaging techniques are paramount to accurately assess these bone dimensions and anatomical relationships [2]. Cone-beam computed tomography (CBCT) has emerged as the preferred imaging modality for dental implant planning due to its ability to provide three-dimensional (3D) imaging of the maxillofacial region at a lower radiation dose compared to conventional medical CT scans [3]. CBCT is recognized for its accuracy in assessing bone volume, identifying vital anatomical structures, and ensuring that implants are positioned safely and effectively [4]. However, the accuracy of CBCT-derived measurements depends not only on the imaging device but also on the software used to interpret the CBCT data [5].

Numerous software programs are available to process CBCT data for linear alveolar bone measurements, each utilizing different algorithms and measurement tools. These software platforms often vary in their user interfaces, functionalities, and image-processing capabilities, which can influence the precision and reliability of measurements [6]. While CBCT itself has been proven to provide accurate volumetric data [7], the influence of software variability on the accuracy of linear bone measurements has not been thoroughly evaluated.

Previous studies have highlighted the importance of precise measurement tools in clinical decision-making, as inaccurate measurements can lead to poor implant placement outcomes [8]. The primary aim of this study was to assess the accuracy of linear measurements for the placement of dental implants using three imaging software (CareStream (Carestream Health, Rochester, New York, United States), RadiAnt (Medixant, Poznan, Poland), and iRYS (Cefla s.c., Imola, Italy)) in CBCT scan of dry goat mandible. Specifically, this research seeks to determine whether any significant discrepancies exist among these software platforms in their ability to accurately measure bone dimensions. By evaluating the accuracy of these software platforms, this study aims to provide clinicians with evidence-based recommendations for selecting the most reliable tools for implant planning, thereby improving overall patient outcomes.

CBCT scans can also help clinicians assess anatomical variations in the mandible, particularly the inferior alveolar canal (IAC) and mental nerve region, reducing the risk of nerve damage during implant placement. One common complication is injury to the inferior alveolar nerve (IAN), which may result in paraesthesia, numbness, or nerve degeneration, especially in elderly or female patients.

Given the rapid advancements in digital imaging technology, it is essential to continuously assess the performance of the software used in conjunction with CBCT for implant planning. Understanding the strengths and limitations of these tools can significantly enhance preoperative planning, reduce surgical risks, and ensure the success of implant procedures in the long term [9]. While various software programs have tested CBCT's linear measurement accuracy, no other study at the time of writing this, to the best of our knowledge, has compared the accuracy of CareStream, RadiAnt, and iRYs software for implant planning. This study aims to compare the accuracy of these programs against gold standard bone measurements.

## Materials and methods

This study was carried out in the Department of Oral Medicine and Radiology, Kalinga Institute of Dental Sciences, Bhubaneswar, Odisha, India. It was approved by the Institutional Ethics Committee of Kalinga Institute of Medical Sciences (protocol number KIIT/KIMS/IEC/1291/2023). A total of 27 dry goat mandibles were procured from a vendor registered under Bhubaneswar Municipal Corporation (Rupali Vending zone, South East ward 35 and 40), in Bhubaneswar, for the study.

Inclusion and exclusion criteria

Intact dry goat mandibles with completely dentate arches were included in this study. Any mandible with visible edentulous space, fracture or fragmentation, and bony defects were excluded from the study.

Sample size

The sample size was calculated using formula:  $n=\frac{z(\frac{&alpha;}{2}).2(p(1&minus;p))}{d^{2}}$ where n is the sample size, zis the level of confidence according to the standard normal distribution, p is the estimated proportion of the population that presents the characteristic, and d is tolerated margin of error. The sample size needed to detect an error of 0.3 mm (for a study power of 0.9 and a=0.05) was calculated as 27 samples.

Equipment and process

The Gold Standard: Physical Measurements using Digital Vernier Callipers

**Figure 1:**
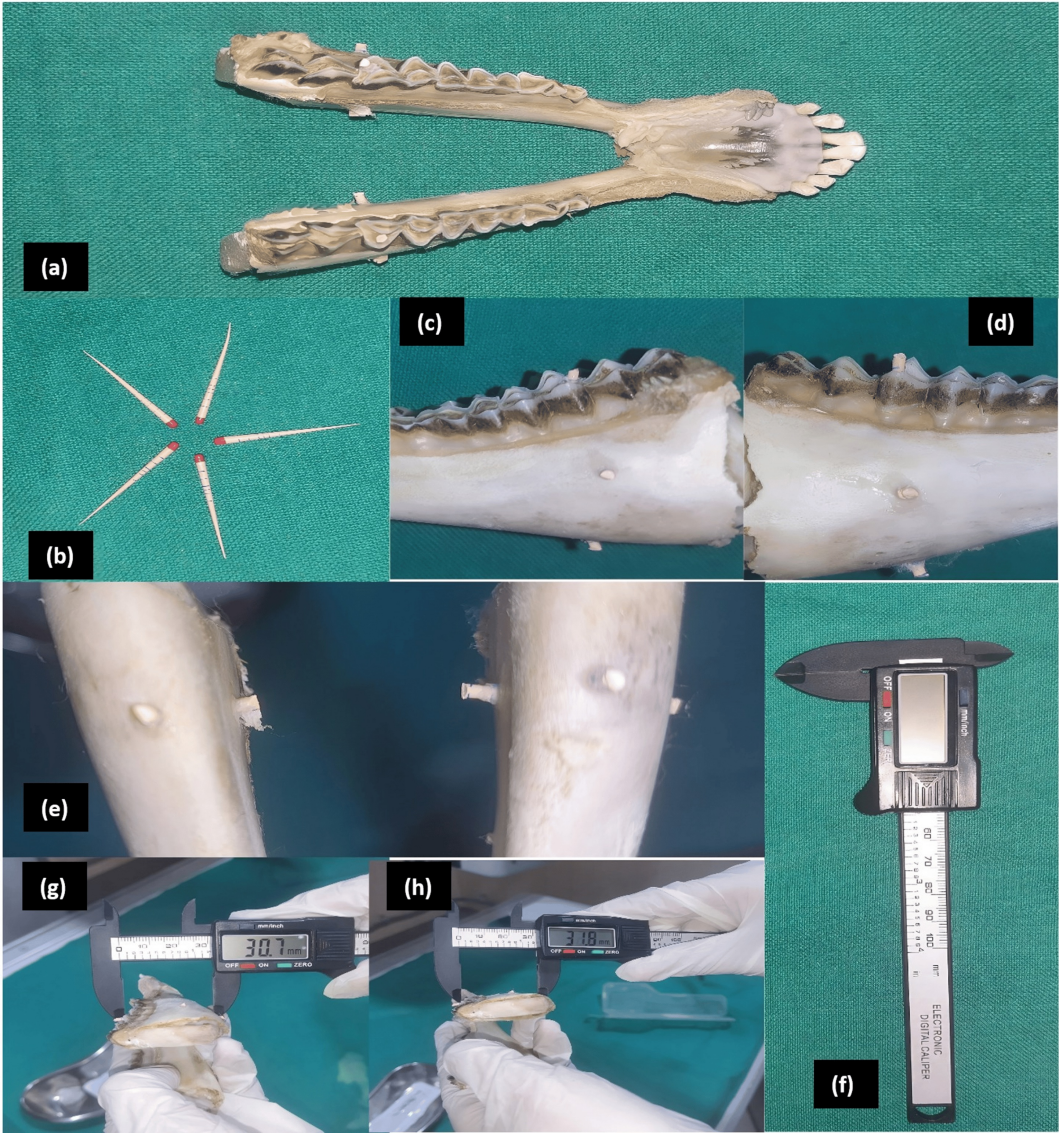
Preparation of the dry goat mandibles and physical measurements (a) A dry goat mandible; (b) Gutta-percha fiducial markers used for delineating the mandible; (c, d) Fiducial markers placed at the region of a single posterior tooth on one side and similar markers were placed on the contralateral tooth; (e) Fiducial marker on the inferior border of the mandible at the region of the posterior teeth; (f) Digital Vernier calipers used to take physical measurements; (g, h) Physical measurements being performed on two separate samples

**Figure 2:**
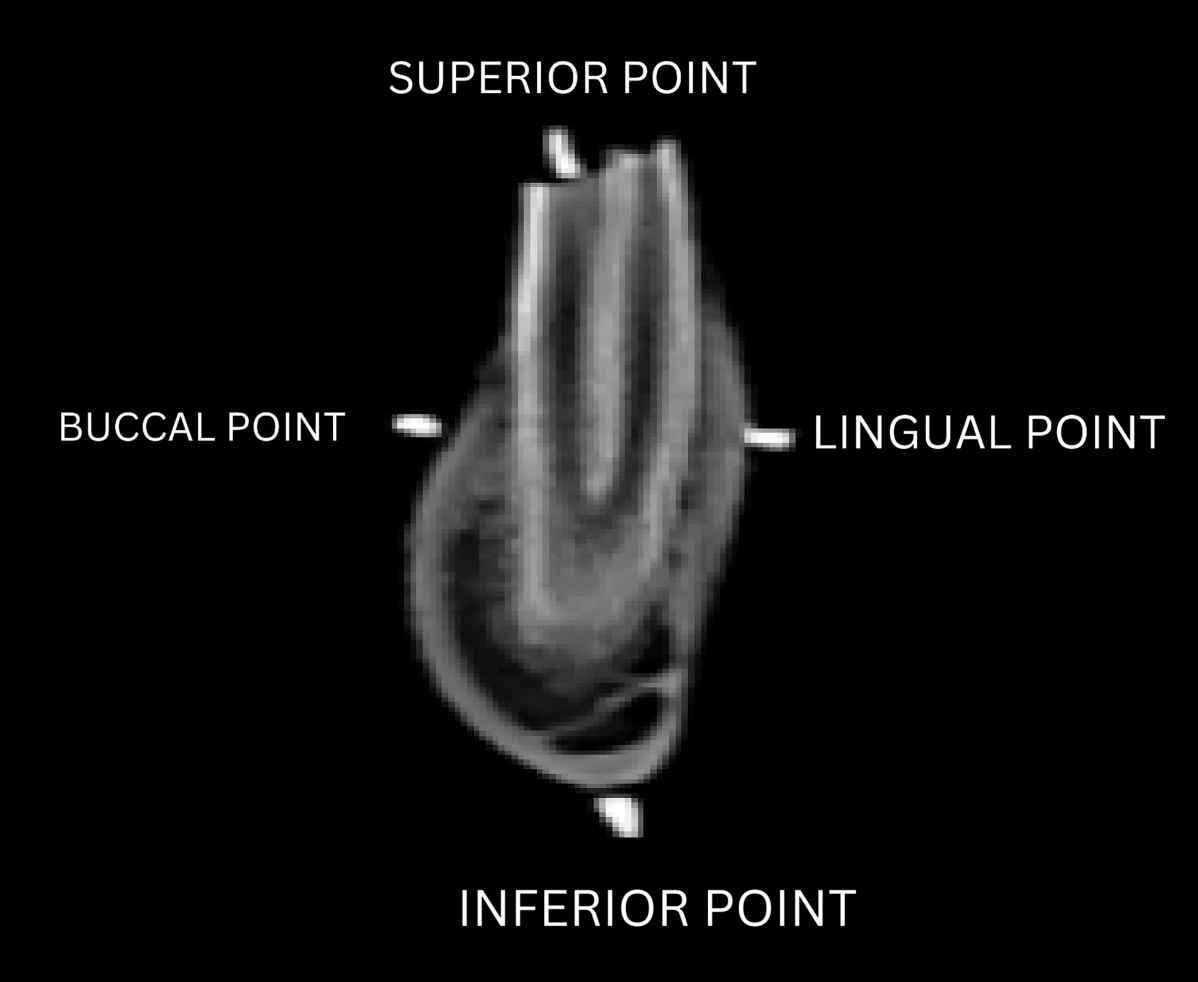
Cross-section of the site of interest taken from the CBCT scan showing the delineation of the mandible The superior and inferior points mark the path of measurement for the mandibular height. The buccal and lingual points mark the path of measurement for the mandibular width. CBCT: cone-beam computed tomography

Radio-opaque fiducial markers in the form of trimmed gutta-percha points (Size #20, Taper 0.6) were placed on the most prominent parts of the first molar region of the dry goat mandibles to delineate sample sites and measurement paths (Figure 1a-1e). The markers were placed on a single posterior tooth bilaterally at the highest point of the occlusal surface of the tooth superiorly and a corroborating marker was placed on the inferior border of the mandible to delineate the height. The prominences of the buccal and lingual cortical plates were marked to delineate the width. The position of the fiducial markers relative to the sample sites and the position and path of the linear measurements are demonstrated in Figure 2.

Digital vernier calipers: A carbon fiber composite digital calipers with a resolution of 0.1 mm/0.01”, Accuracy of +0.1 mm/0.01”, and power of 1.5 V was used to take the physical measurements of the dry goat mandibles at the region of a single posterior tooth bilaterally (Figure 1f-1h). Four sample measurements were obtained by recording the height and width of the mandible with teeth at the bilateral posterior tooth regions from the 27 goat mandibles constituting a total of 108 measurements. Each set of measurements was repeated thrice and performed by two examiners (AN, SM) to ascertain reliability.

Scanning the Mandibles

**Figure 3:**
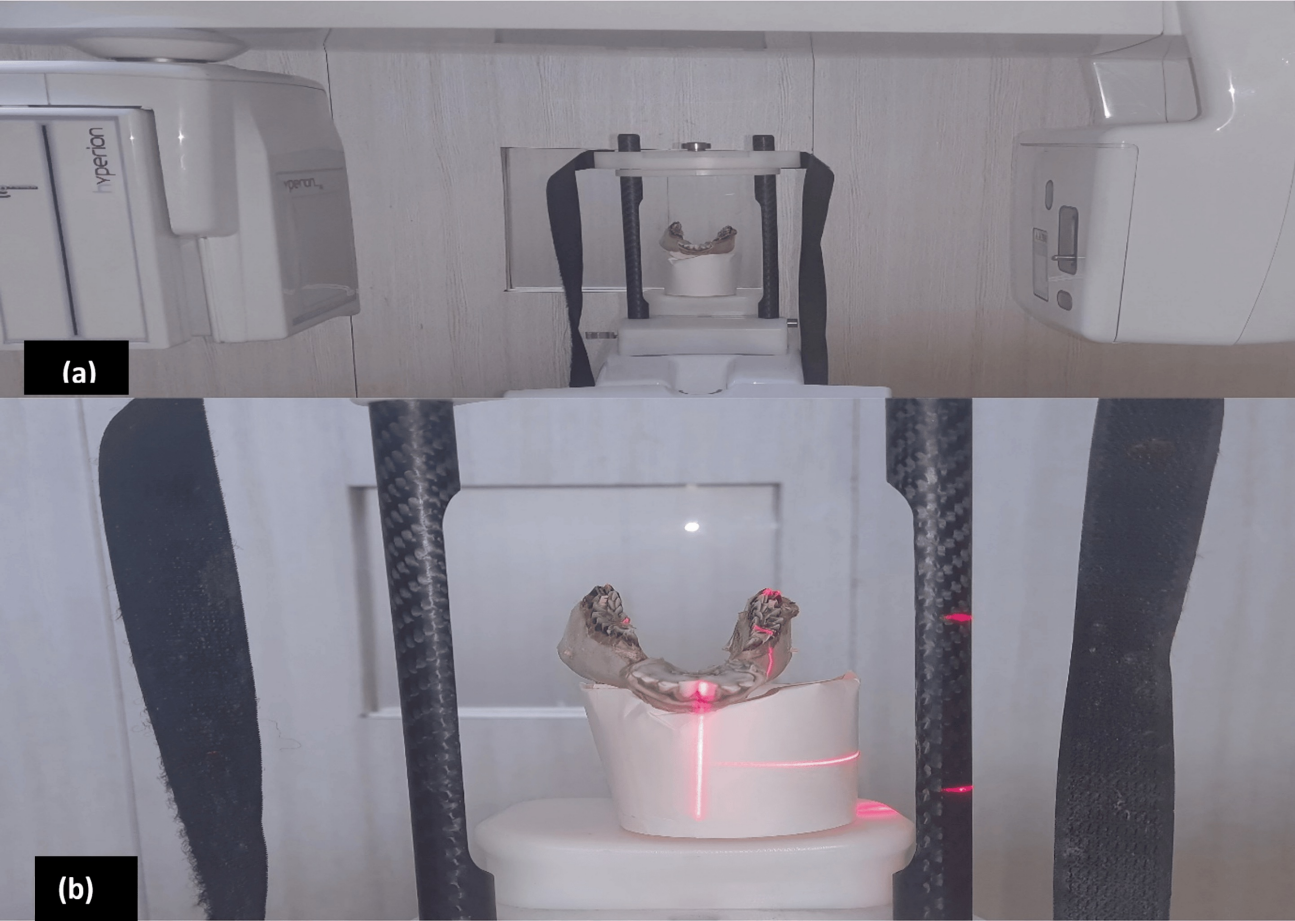
Scanning of the mandibles (a) Dry mandible placed on Styrofoam platform for scan; (b) Mandible ready for scan on MyRaY Hyperion X9 3-in-1 imaging device (Cefla s.c., Imola, Italy) in three-dimensional scan setting.

The mandibles were placed on a Styrofoam (DuPont de Nemours, Inc., Wilmington, Delaware, United States) platform to stabilize on the chin rest to reach the focal trough. The CBCT exposure parameters used were 10 mA, 90 kVp, and 40 seconds of exposure time. The placement of the mandibles is shown in Figure 3.

Computer System

A desktop computer equipped with an 11th Gen Intel Core i7 processor (2.80 GHz), and integrated iRIS_XE_ graphics (Intel Corporation, Santa Clara, California, United States) was used to process and analyze the CBCT data. The CBCT dataset for each mandible was exported in DICOM (digital imaging and communication in medicine) format to an external hard drive. The datasets were then downloaded to the hard drive of a computer in which the software programs were installed.

Software

Each CBCT dataset was viewed with the three test software programs on the same computer and using the same display settings: CareStream 3D Imaging v3.10.8, RadiAnt DICOM Viewer, version 5.5.0, and iRYs version 8.0.

**Figure 4:**
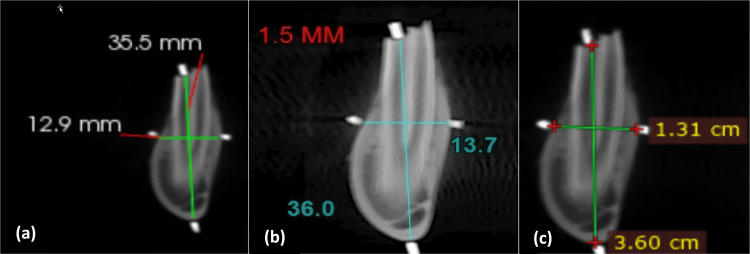
Mandibular height and width measurements done using (a) Carestream software* (in mm), (b) iRYS software** (in mm), and (c) RadiAnt software*** (in cm) *Carestream Health, Rochester, New York, United States; **Cefla s.c., Imola, Italy; ***Medixant, Poznan, Poland

For CareStream andiRYs, the multi-planar tool was used to trace the jaw on the axial section, and corroborating transverse cross-sections were automatically generated by the program according to the arch tracing. For RadiAnt, the orthogonal sectional planes were individually shifted and tilted by adjusting the reformatting lines corresponding to the sectional images. So, the lines were aligned precisely along the long axis of the alveolar bone in the display images of the sample sites. The height and width of each site were measured using linear measurement tools of the software at the delineated marks. The iRYS and CareStream measurements were recorded in millimeters while the measurements with the RadiAnt software were recorded in centimeters, which were converted to millimeters for the sake of lucidity in comparison. The procedure is demonstrated in Figure 4. All the measurements were entered into a singular data sheet on Microsoft Excel 2016 (Microsoft Corporation, Redmond, Washington, United States) as shown in Table 1.

**Table 1:** Datasheet with mandibular measurements done with Vernier calipers (column: Gold standard), iRYS*, CareStream**, and RadiAnt*** MH and MW were recorded in mm across all domains. In iRYS, CareStream and RadiAnt, the MH and MW were calculated at three slice thicknesses: 0.15 mm, 1.05 mm, and 1.50 mm. *CareStream 3D Imaging v3.10.8 (Carestream Health, Rochester, New York, United States); **RadiAnt DICOM Viewer, version 5.5.0 (Medixant, Poznan, Poland), and iRYs version 8.0 (Cefla s.c., Imola, Italy). MH: mandibular height; MW: mandibular width; DICOM: digital imaging and communication in medicine

Sample	Measurements (in mm)	Gold Standard		iRYS						CareStream						RadiAnt					
		Right	Left	Right			Left			Right			Left			Right			Left		
																										0.15mm	1.05mm	1.50mm	0.15mm	1.05mm	1.50mm	0.15mm	1.05mm	1.50mm	0.15mm	1.05mm	1.50mm	0.15mm	1.05mm	1.50mm	0.15mm	1.05mm	1.50mm
																																												Mandible 1	MH	36.4	36.3	35.9	35.8	36.2	36.5	36.3	36.4	35.8	35.8	35.9	36.3	36.1	35.9	36.5	36	36.1	36.5	36	36.1
MW	14.3	14.6	14.1	14.3	14.3	14.5	14.4	14.5	14.3	14.7	14.5	14	14.1	14.1	14.4	14.5	14.6	14.4	14.5	14.6																								
Mandible 2	MH	32.5	32.2	31.8	32.3	31.8	32.4	32.4	32.4	35.5	34.9	35.3	32	32.2	32.3	36.1	36	36	36.1	36.2	36.1																						
	MW	12.9	12.7	12.2	12.4	12.1	11.6	11.6	12.4	12.2	12.9	12.9	12.4	12.7	12.6	13.2	13.1	13.1	13.2	13.2	13.2																						
Mandible 3	MH	30.7	31.2	30.5	30.9	31	31.5	30.9	31.4	30.8	31	31.1	30.7	31	30.6	31.2	31.3	30.8	31	31.1	30.7
	MW	14.2	14.6	14.3	14.5	14.4	14.7	14.5	14.3	14.4	14.7	14.3	14.5	14.3	14.4	14.6	14.5	14.4	14.7	14.3	14.5
Mandible 4	MH	34.2	34.1	34	33.8	34.3	34.4	34.5	34.2	34	34.1	33.9	34.3	34.2	34	34.4	34.1	34	34.1	33.9	34.3
	MW	13.6	13.7	13.5	13.6	13.8	13.6	13.7	13.8	13.5	13.7	13.6	13.5	13.7	13.6	13.8	13.7	13.5	13.7	13.6	13.5
Mandible 5	MH	36.8	36.8	36.5	36.7	36.6	36.9	36.7	36.8	36.4	36.6	36.5	36.9	36.8	36.5	36.9	36.7	36.4	36.6	36.5	36.9
	MW	14.9	14.7	14.8	14.6	14.7	14.9	14.6	14.8	14.7	14.8	14.7	14.6	14.9	14.7	14.9	14.8	14.7	14.8	14.7	14.6
Mandible 6	MH	28.9	30.6	28.8	30.3	29.3	30.4	28.7	30.5	29.2	30.2	28.9	30.6	28.8	30.4	29.1	30.3	29.2	30.2	28.9	30.6
	MW	12.3	12.1	12	12.2	12.3	12.1	12.4	12.2	12.3	12	12.1	12.1	12.3	12.2	12.1	12.4	12.3	12	12.1	12.1
Mandible 7	MH	32	32.1	30.5	30.9	31	31.5	30.9	31.4	30.8	31.2	31.1	30.7	31	30.6	31.2	31.3	30.8	31	31.1	30.7
	MW	13.5	13.6	13.4	13.5	13.6	13.4	13.7	13.6	13.5	13.4	13.5	13.6	13.4	13.5	13.7	13.6	13.5	13.6	13.4	13.5
Mandible 8	MH	35.5	35.7	35.6	35.8	35.7	35.5	35.6	35.7	35.4	35.6	35.7	35.8	35.5	35.7	35.9	35.6	35.5	35.8	35.5	35.7
	MW	15	14.8	14.9	14.7	15.1	14.9	15	14.8	15.1	14.9	15	14.8	14.9	15	14.9	15.1	14.8	14.8	14.9	15
Mandible 9	MH	30.2	30.4	30.1	30.3	30.2	30.4	30	30.3	30.5	30.2	30.1	30.4	30.3	30.1	30.2	30.5	30.2	30.4	30.3	30.1
	MW	12.8	12.9	12.7	12.8	12.6	12.7	12.9	12.8	12.6	12.8	12.7	12.9	12.6	12.8	12.7	12.8	12.7	12.9	12.6	12.8
Mandible 10	MH	34.8	34.9	34.6	34.8	34.9	35	34.8	34.9	35	34.6	34.9	34.7	35	34.8	34.9	35.1	34.8	34.7	35	34.8
	MW	14.1	14.3	14.2	14.4	14.3	14.1	14.4	14.3	14.1	14.2	14.3	14.2	14.3	14.4	14.3	14.2	14.1	14.2	14.3	14.4
Mandible 11	MH	36.2	36.3	36.1	36	36.2	36.3	36	36.2	36.1	36.4	36.3	36.1	36.2	36.3	36.4	36	36.2	36.1	36.2	36.3
	MW	15.2	15.1	14.9	15.2	15.1	15.2	15.1	15.3	15.1	15.1	15.2	14.8	15.1	15.4	15.1	15.2	15	15	15.1	15
Mandible 12	MH	33	33.1	33.2	33	33.1	33.2	33	33.1	33.2	33.1	32.9	33.2	33.1	33	33.2	33.1	33.2	33.2	33.1	33
	MW	13.9	13.8	13.7	13.9	13.8	13.7	13.9	13.8	13.9	13.8	13.7	13.8	13.9	13.7	13.9	13.8	13.7	13.8	13.9	13.7
Mandible 13	MH	29.6	29.8	29.5	29.7	29.6	29.8	29.7	29.5	29.7	29.5	29.6	29.7	29.5	29.6	29.7	29.6	29.8	29.7	29.5	29.6
	MW	12.6	12.5	12.7	12.6	12.5	12.7	12.6	12.5	12.6	12.7	12.5	12.6	12.5	12.7	12.6	12.5	12.6	12.6	12.5	12.7
Mandible 14	MH	32	32.1	32	32.1	31.9	31.8	32.1	31.8	31.9	32.1	31.9	32	32.1	31.9	32.2	32.1	31.9	32	32.1	31.9
	MW	13.5	13.6	13.2	13.3	13.4	13.4	13.5	13.5	13.2	13.3	13.4	13.4	13.5	13.5	13.4	13.5	13.5	13.2	13.3	13.4
Mandible 15	MH	35.5	35.7	35	35.3	35.4	35.6	35.7	35.7	35.1	35.1	35.1	35.8	35.8	35.7	35	35.3	35.4	35.6	35.7	35.7
	MW	15	14.8	14.8	14.9	14.9	14.7	14.5	14.7	14.5	14.8	14.9	14.9	14.7	14.5	14.7	14.7	14.5	14.8	14.8	14.9
Mandible 16	MH	30.2	30.4	30.1	30.2	30.4	30	30.4	30.4	30	30.4	30.4	30	30.4	30.4	30	30.4	30.4	30	30.4	30.4
	MW	12.8	12.9	12.4	12.5	12.7	12.8	13	13	12.7	12.7	12.8	12.8	12.7	12.9	12.6	12.7	12.7	12.9	12.9	13.1
Mandible 17	MH	36.4	36.3	35.9	35.8	36.2	36.5	36.3	36.4	35.8	35.8	35.9	36.3	36.1	35.9	36.5	36	36.1	36.5	36	36.1
	MW	14.3	14.6	14.1	14.3	14.3	14.5	14.4	14.5	14.3	14.7	14.5	14	14.1	14.1	14.4	14.5	14.6	14.4	14.5	14.6
Mandible 18	MH	32.5	32.2	31.8	32.3	31.8	32.4	32.4	32.4	35.5	34.9	35.3	32	32.2	32.3	36.1	36	36	36.1	36.2	36.1
	MW	12.9	12.7	12.2	12.4	12.1	11.6	11.6	12.4	12.2	12.9	12.9	12.4	12.7	12.6	13.2	13.1	13.1	13.2	13.2	13.2
Mandible 19	MH	30.7	31.2	30.5	30.9	31	31.5	30.9	31.4	30.8	31	31.1	30.7	31	30.6	31.2	31.3	30.8	31	31.1	30.7
	MW	14.2	14.6	14.3	14.5	14.4	14.7	14.5	14.3	14.4	14.7	14.3	14.5	14.3	14.4	14.6	14.5	14.4	14.7	14.3	14.5
Mandible 20	MH	34.2	34.1	34	33.8	34.3	34.4	34.5	34.2	34	34.1	33.9	34.3	34.2	34	34.4	34.1	34.2	34.1	33.9	34.3
	MW	13.6	13.7	13.5	13.6	13.8	13.6	13.7	13.8	13.5	13.7	13.6	13.5	13.7	13.6	13.8	13.7	13.5	13.7	13.6	13.5
Mandible 21	MH	36.8	36.8	36.5	36.7	36.6	36.9	36.7	36.8	36.4	36.6	36.5	36.9	36.8	36.5	36.9	36.7	36.4	36.6	36.5	36.9
	MW	14.9	14.7	14.8	14.6	14.7	14.9	14.6	14.8	14.7	14.8	14.7	14.6	14.9	14.7	14.9	14.8	14.7	14.8	14.7	14.6
Mandible 22	MH	28.9	30.6	28.8	30.3	29	30.4	28.7	30.5	29.2	30.2	28.9	30.6	28.8	30.4	29.1	30.3	29.2	30.2	28.9	30.6
	MW	12.6	12.1	12	12.2	12.3	12.1	12.4	12.2	12.3	12	12.1	12.1	12.3	12.2	12.1	12.4	12.3	12	12.1	12.1
Mandible 23	MH	32	32.1	30.5	30.9	31	31.5	30.9	31.4	30.8	31	31.1	30.7	31	30.6	31.2	31.3	30.8	31	31.1	30.7
	MW	13.5	13.6	13.4	13.5	13.6	13.4	13.7	13.6	13.5	13.4	13.5	13.6	13.4	13.5	13.7	13.6	13.5	13.6	13.4	13.5
Mandible 24	MH	35.5	35.7	35.6	35.8	35.7	35.5	35.6	35.7	35.4	35.6	35.7	35.8	35.5	35.7	35.9	35.6	35.5	35.8	35.5	35.7
	MW	15	14.8	14.9	14.7	15.1	14.9	15	14.8	15.1	14.9	15.2	14.8	14.9	15	14.9	15.1	14.8	14.8	14.9	15
Mandible 25	MH	30.2	30.4	30.1	30.3	30.2	30.4	30	30.3	30.5	30.2	30.1	30.4	30.3	30.1	30.2	30.5	30.2	30.4	30.3	30.1
	MW	12.8	12.9	12.7	12.8	12.6	12.7	12.9	12.8	12.6	12.8	12.7	12.9	12.6	12.8	12.7	12.8	12.7	12.9	12.6	12.8
Mandible 26	MH	34.8	34.9	34.6	34.8	34.9	35	34.8	34.9	35	34.6	34.9	34.7	35	34.8	34.9	35.1	34.8	34.7	35	34.8
	MW	14.1	14.3	14.2	14.4	14.3	14.1	14.4	14.3	14.1	14.2	14.3	14.2	14.3	14.4	14.3	14.2	14.1	14.2	14.3	14.4
Mandible 27	MH	36.2	36.3	36.1	36	36.2	36.3	36	36.2	36.1	36.4	36.3	36.1	36.2	36.3	36.4	36	36.2	36.1	36.2	36.3
	MW	15.2	15.1	15.2	15.2	15.1	15.1	15.1	15.3	15	15.1	15.2	15	15.1	15.2	15.1	15.2	15.1	15	15.1	15.1

Thirty measurements out of the 108 measurements were selected for reliability testing. Five sample sites were randomly selected for each software program using an online random number generator and the height and width measurements at each site were used for reliability testing. The measurements were recorded by the same examiners.

Comparison and data analysis

The accuracy of the linear measurements from each software was compared to the gold standard measurements taken with the digital Vernier calipers (also referred to as the control). Absolute measurement errors (in mm) were calculated for each software program. Descriptive and inferential statistical analyses were conducted to evaluate any significant differences between the software programs in terms of measurement accuracy using the ANOVA test. The multigroup comparison was done using Tukey's Honestly Significant Difference (HSD) [10].

## Results

Across all error thresholds, the software programs demonstrated high accuracy with minimal variation from the control values. iRYS and RadiAnt consistently produced results close to the control, with iRYS showing slightly better accuracy at lower error thresholds and RadiAnt performing well at higher thresholds.

The iRYS software produced a mean mandibular height of 32.91 mm (right) and 33.41 mm (left), 33.17 mm (right) and 33.13 mm (left), and 33.13 mm (right) and 33.37 mm (left) for error thresholds of 0.15 mm, 1.05 mm, and 1.5 mm slice thickness, respectively. Upon comparison with CareStream, RadiAnt, and control, a probability value (p-value) of 0.856-0.988 was obtained suggesting no significant difference. The mean mandibular width was marked up at 13.67 mm (right) and 13.73 mm (left), 13.76 mm (right) and 13.78 mm (left), and 13.77mm (right) and 13.82 mm (left) for error thresholds of 0.15 mm, 1.05 mm, and 1.5 mm slice thickness, respectively, showing measurements close to the control, especially at 0.15 mm and 1.50 mm thickness. Upon comparison with CareStream, RadiAnt, and control, a p-value of 0.847-0.995 was obtained suggesting no significant difference.

The CareStream software produced a mean mandibular height of 33.29 mm (right) and 33.25 mm (left), 33.38 mm (right) and 33.15 mm (left), and 33.31 mm (right) and 33.15 mm (left) for error thresholds of 0.15 mm, 1.05 mm, and 1.5 mm slice thickness, respectively. Upon comparison with iRYS, RadiAnt, and control, a p-value of 0.856-0.988 was obtained suggesting no significant difference. The mean mandibular width was marked up at 13.72 mm (both right and left), 13.84 mm (right) and 13.77 mm (left), and 13.82 mm (right) and 13.80 mm (left) for error thresholds of 0.15 mm, 1.05 mm, and 1.5 mm slice thickness, respectively. The measurements were slightly lower than the control but within the acceptable range. Upon comparison with iRYS, RadiAnt, and control, a p-value of 0.847-0.995 was obtained suggesting no significant difference.

The RadiAnt software produced a mean mandibular height of 33.54 mm (both right and left), 33.57 mm (right) and 33.42 mm (left), and 33.37 mm (right) and 33.53 mm (left) for error thresholds of 0.15 mm, 1.05 mm, and 1.5 mm slice thickness, respectively. Upon comparison with iRYS, CareStream, and control, a p-value of 0.856-0.988 was obtained suggesting no significant difference. The mean mandibular width was marked up at 13.87 mm (right) and 13.85 mm (left), 13.89 mm (right) and 13.81 mm (left), and 13.80 mm (right) and 13.86 mm (left) for error thresholds of 0.15 mm, 1.05 mm, and 1.5 mm slice thickness, respectively. The measurements were slightly overestimated at lower thresholds while matching closely to the control at 1.05 mm thickness. Upon comparison with CareStream and control, a p-value of 0.847-0.995 was obtained suggesting no significant difference.

The standard deviations across the software programs and control were small, indicating consistent measurements, with minor variations among the software programs. RadiAnt generally shows slightly higher accuracy at higher error thresholds (1.05 mm and 1.50 mm), while iRYS and CareStream were consistent but slightly lower than the control values. All p-values are above the commonly accepted threshold of 0.05, suggesting that any observed differences were likely due to chance rather than meaningful variations in performance. This implies that all systems may perform similarly under the conditions tested. Further research may be needed to explore additional factors or to analyze a larger sample size to detect potential differences. The findings have been demonstrated in tabular form of descriptive statistics (Tables 2-5) and multigroup comparison of the groups (Tables 6-9) for mandibular height and width on the right and left sides.

**Table 2:** Descriptive statistics for mandibular height on the right side The mandibular height was recorded in millimeters (mm). The measurements were done at slice thicknesses of 0.15 mm, 1.05 mm, and 1.50 mm. The descriptive and inferential statistical analyses were done using ANOVA test.

		N	Mean	SD	Minimum	Maximum	F Score	P-value
0.15 mm	iRYS	27	32.911	2.6793	28.8	36.5	.257	.856
	CareStream	27	33.293	2.6019	29.2	36.4		
	RadiAnt	27	33.544	2.7794	29.1	36.9		
	Control	27	33.211	2.6470	28.9	36.8		
1.05 mm	iRYS	27	33.167	2.4523	29.7	36.7	.141	.935
	CareStream	27	33.378	2.4806	29.5	36.6		
	RadiAnt	27	33.567	2.4849	29.6	36.7		
	Control	27	33.211	2.6470	28.9	36.8		
1.50 mm	iRYS	27	33.130	2.6396	29.0	36.6	.042	.988
	CareStream	27	33.307	2.6558	28.9	36.5		
	RadiAnt	27	33.367	2.6785	29.2	36.4		
	Control	27	33.211	2.6470	28.9	36.8		

**Table 3:** Descriptive statistics for mandibular height for the left side The mandibular height was recorded in millimeters (mm). The measurements were done at slice thicknesses of 0.15 mm, 1.05 mm, and 1.50 mm. The descriptive and inferential statistical analyses were done using ANOVA test.

		N	Mean	SD	Minimum	Maximum	F Score	P-value
0.15 mm	iRYS	27	33.407	2.4875	29.8	36.9	.060	.981
	CareStream	27	33.248	2.5688	29.7	36.9		
	RadiAnt	27	33.537	2.6154	29.7	36.6		
	Control	27	33.419	2.4136	29.8	36.8		
1.05 mm	iRYS	27	33.130	2.7054	28.7	36.7	.099	.960
	CareStream	27	33.152	2.6757	28.8	36.8		
	RadiAnt	27	33.415	2.7082	28.9	36.5		
	Control	27	33.419	2.4136	29.8	36.8		
1.50 mm	iRYS	27	33.367	2.4754	29.5	36.8	.107	.956
	CareStream	27	33.148	2.5325	29.6	36.5		
	RadiAnt	27	33.526	2.6598	29.6	36.9		
	Control	27	33.419	2.4136	29.8	36.8		

**Table 4:** Descriptive statistics for mandibular width for the right side The mandibular width was recorded in millimeters (mm). The measurements were done at slice thicknesses of 0.15 mm, 1.05 mm, and 1.50 mm. The descriptive and inferential statistical analyses were done using ANOVA test.

		N	Mean	SD	Minimum	Maximum	F Score	p-value
0.15 mm	iRYS	27	13.670	1.0227	12.0	15.2	.270	.847
	CareStream	27	13.719	.9946	12.2	15.1		
	RadiAnt	27	13.874	.9481	12.1	15.1		
	Control	27	13.841	.9141	12.3	15.2		
1.05 mm	iRYS	27	13.763	.9783	12.2	15.2	.081	.970
	CareStream	27	13.841	.9889	12.0	15.1		
	RadiAnt	27	13.889	.9233	12.4	15.2		
	Control	27	13.841	.9141	12.3	15.2		
1.50 mm	iRYS	27	13.774	1.0302	12.1	15.1	.024	.995
	CareStream	27	13.819	.9818	12.1	15.2		
	RadiAnt	27	13.796	.8864	12.3	15.1		
	Control	27	13.841	.9141	12.3	15.2		

**Table 5:** Descriptive statistics for Mandibular Width on the Left Side The mandibular width was recorded in millimeters (mm). The measurements were done at slice thicknesses of 0.15 mm, 1.05 mm, and 1.50 mm. The descriptive and inferential statistical analyses were done using ANOVA test.

		N	Mean	SD	Minimum	Maximum	F Score	P-value
0.15 mm	iRYS	27	13.726	1.1196	11.6	15.2	.139	.936
	CareStream	27	13.722	.9267	12.1	15.0		
	RadiAnt	27	13.848	.9200	12.0	15.0		
	Control	27	13.844	.9500	12.1	15.1		
1.05 mm	iRYS	27	13.781	1.0355	11.6	15.1	.029	.993
	CareStream	27	13.774	.9473	12.3	15.1		
	RadiAnt	27	13.807	.9393	12.1	15.1		
	Control	27	13.844	.9500	12.1	15.1		
1.50 mm	iRYS	27	13.819	.9771	12.2	15.3	.026	.994
	CareStream	27	13.796	.9509	12.2	15.4		
	RadiAnt	27	13.863	.9220	12.1	15.1		
	Control	27	13.844	.9500	12.1	15.1		

**Table 6:** Multiple group comparison for the right side mandibular height The mandibular height was recorded in millimeters (mm). The measurements were done at slice thicknesses of 0.15 mm, 1.05 mm, and 1.50 mm. The comparison was done using Tukey's Honestly Significant Difference (HSD).

Dependent Variable	I Group	J Group	Mean Difference (I-J)	Std. Error	P-value	95% Confidence Interval	
						Lower Bound	Upper Bound
0.15 mm	iRYS	CareStream	-.3815	.7288	.953	-2.284	1.521
		RadiAnt	-.6333	.7288	.821	-2.536	1.270
		Control	-.3000	.7288	.976	-2.203	1.603
	CareStream	RadiAnt	-.2519	.7288	.986	-2.155	1.651
		Control	.0815	.7288	.999	-1.821	1.984
	RadiAnt	Control	.3333	.7288	.968	-1.570	2.236
1.05 mm	iRYS	CareStream	-.2111	.6851	.990	-2.000	1.578
		RadiAnt	-.4000	.6851	.937	-2.189	1.389
		Control	-.0444	.6851	1.000	-1.833	1.744
	CareStream	RadiAnt	-.1889	.6851	.993	-1.978	1.600
		Control	.1667	.6851	.995	-1.622	1.956
	RadiAnt	Control	.3556	.6851	.954	-1.433	2.144
1.50 mm	iRYS	CareStream	-.1778	.7227	.995	-2.065	1.709
		RadiAnt	-.2370	.7227	.988	-2.124	1.650
		Control	-.0815	.7227	.999	-1.968	1.805
	CareStream	RadiAnt	-.0593	.7227	1.000	-1.946	1.828
		Control	.0963	.7227	.999	-1.791	1.983
	RadiAnt	Control	.1556	.7227	.996	-1.731	2.042

**Table 7:** Multiple group comparison for the group for the left side mandibular height. The mandibular height was recorded in millimeter (mm). The measurements were done at slice thicknesses of 0.15 mm, 1.05 mm, and 1.50 mm. The comparison was done using Tukey's Honestly Significant Difference (HSD) test.

Dependent Variable	I Group	J Group	Mean Difference (I-J)	Std. Error	P-value	95% Confidence Interval	
						Lower Bound	Upper Bound
0.15 mm	iRYS	CareStream	.1593	.6865	.996	-1.633	1.952
		RadiAnt	-.1296	.6865	.998	-1.922	1.663
		Control	-.0111	.6865	1.000	-1.804	1.781
	CareStream	RadiAnt	-.2889	.6865	.975	-2.081	1.504
		Control	-.1704	.6865	.995	-1.963	1.622
	RadiAnt	Control	.1185	.6865	.998	-1.674	1.911
1.05 mm	iRYS	CareStream	-.0222	.7154	1.000	-1.890	1.846
		RadiAnt	-.2852	.7154	.978	-2.153	1.583
		Control	-.2889	.7154	.978	-2.157	1.579
	CareStream	RadiAnt	-.2630	.7154	.983	-2.131	1.605
		Control	-.2667	.7154	.982	-2.135	1.601
	RadiAnt	Control	-.0037	.7154	1.000	-1.872	1.864
1.50 mm	iRYS	CareStream	.2185	.6864	.989	-1.574	2.011
		RadiAnt	-.1593	.6864	.996	-1.951	1.633
		Control	-.0519	.6864	1.000	-1.844	1.740
	CareStream	RadiAnt	-.3778	.6864	.946	-2.170	1.414
		Control	-.2704	.6864	.979	-2.063	1.522
	RadiAnt	Control	.1074	.6864	.999	-1.685	1.900

**Table 8:** Multiple group comparison for the group on the right side mandibular width. The mandibular width was recorded in millimeters. The measurements were done at slice thicknesses of 0.15 mm, 1.05 mm, and 1.50 mm. The comparison test was conducted using Tukey's Honestly Significant Difference (HSD) test.

Dependent Variable	I Group	J Group	Mean Difference (I-J)	Std. Error	P-value	95% Confidence Interval	
						Lower Bound	Upper Bound
0.15 mm	iRYS	CareStream	-.0481	.2642	.998	-.738	.642
		RadiAnt	-.2037	.2642	.867	-.894	.486
		Control	-.1704	.2642	.917	-.860	.520
	CareStream	RadiAnt	-.1556	.2642	.935	-.845	.534
		Control	-.1222	.2642	.967	-.812	.568
	RadiAnt	Control	.0333	.2642	.999	-.657	.723
1.05 mm	iRYS	CareStream	-.0778	.2590	.991	-.754	.599
		RadiAnt	-.1259	.2590	.962	-.802	.550
		Control	-.0778	.2590	.991	-.754	.599
	CareStream	RadiAnt	-.0481	.2590	.998	-.724	.628
		Control	.0000	.2590	1.000	-.676	.676
	RadiAnt	Control	.0481	.2590	.998	-.628	.724
1.50 mm	iRYS	CareStream	-.0444	.2599	.998	-.723	.634
		RadiAnt	-.0222	.2599	1.000	-.701	.656
		Control	-.0667	.2599	.994	-.745	.612
	CareStream	RadiAnt	.0222	.2599	1.000	-.656	.701
		Control	-.0222	.2599	1.000	-.701	.656
	RadiAnt	Control	-.0444	.2599	.998	-.723	.634

**Table 9:** Multiple group comparison for the group for the left side mandibular width The mandibular width was recorded in millimeters( mm). The measurements were done at slice thicknesses of 0.15 mm, 1.05 mm, and 1.50 mm. The comparison was conducted using Tukey's Honestly Significant Difference (HSD) test.

Dependent Variable	I Group	J Group	Mean Difference (I-J)	Std. Error	P-value	95% Confidence Interval	
						Lower Bound	Upper Bound
0.15 mm	iRYS	CareStream	.0037	.2674	1.000	-.695	.702
		RadiAnt	-.1222	.2674	.968	-.820	.576
		Control	-.1185	.2674	.971	-.817	.580
	CareStream	RadiAnt	-.1259	.2674	.965	-.824	.572
		Control	-.1222	.2674	.968	-.820	.576
	RadiAnt	Control	.0037	.2674	1.000	-.695	.702
1.05 mm	iRYS	CareStream	.0074	.2637	1.000	-.681	.696
		RadiAnt	-.0259	.2637	1.000	-.714	.663
		Control	-.0630	.2637	.995	-.751	.626
	CareStream	RadiAnt	-.0333	.2637	.999	-.722	.655
		Control	-.0704	.2637	.993	-.759	.618
	RadiAnt	Control	-.0370	.2637	.999	-.726	.651
1.50 mm	iRYS	CareStream	.0222	.2586	1.000	-.653	.697
		RadiAnt	-.0444	.2586	.998	-.720	.631
		Control	-.0259	.2586	1.000	-.701	.649
	CareStream	RadiAnt	-.0667	.2586	.994	-.742	.609
		Control	-.0481	.2586	.998	-.723	.627
	RadiAnt	Control	.0185	.2586	1.000	-.657	.694

## Discussion

The present study aimed to assess the accuracy of linear alveolar bone measurements for implant planning using CBCT by comparing three 3D imaging software programs: iRYS, CareStream, and RadiAnt. The findings indicate that iRYS consistently provides measurements closest to the control, especially at lower error thresholds. In contrast, RadiAnt tends to overestimate measurements, while CareStream displays intermediate accuracy regarding measurement values and deviations.

These results are in line with previous research that evaluated the accuracy of various imaging software for dental applications. For instance, a study by Yusof et al. demonstrated that different CBCT software can yield varying degrees of accuracy in linear measurements, significantly influenced by factors such as image resolution and calibration protocols [11]. The consistent performance of iRYS in producing lower deviations from the control corroborated the findings from other studies, which indicated that specific software can achieve higher accuracy in certain measurement contexts as proposed by Luangchana et al. in 2015 [12].

RadiAnt's tendency to overestimate measurements aligns with findings by Carneiro et al. in 2024, who identified systematic biases in certain imaging programs, particularly when assessing cortical bone thickness [13]. The higher measurements observed for RadiAnt suggest that practitioners using this software should exercise caution, especially when precise bone measurements are critical for successful implant placement as proposed by Fokas et al. in 2018 [14]. CareStream's intermediate performance, showing values slightly higher than iRYS but generally lower than RadiAnt, is consistent with literature indicating that while it provides reliable measurements, it may not be as precise as its competitors in specific contexts as proposed by Karkle et al. in 2024 [15]. This consideration is vital for clinicians who must choose software based on their specific measurement needs and the required precision for successful implant outcomes.

The high accuracy of all three software programs suggests their reliability for implant planning, as the measured values were consistently close to the control across various error thresholds. This finding supports the broader literature emphasizing that CBCT imaging, combined with competent software, can provide accurate representations of anatomical structures necessary for effective implant planning [13]. The small standard deviations observed across all programs indicate a high level of consistency in measurements, reinforcing their utility in clinical practice [12]. While iRYS demonstrates superior performance, particularly at lower error thresholds (0.15 mm and 1.50 mm), it is important to note that all software maintained acceptable accuracy levels. This indicates that, despite minor variations, all three programs can be reliably utilized in clinical settings, providing flexibility in software choice based on practitioner preference or specific clinical scenarios [14].

The findings highlight the importance of selecting appropriate imaging software for implant planning. iRYS's closer alignment with control values makes it preferable for situations where precision is paramount. However, clinicians should remain aware of RadiAnt's propensity to overestimate and CareStream's intermediate performance, particularly in challenging anatomical situations where measurement accuracy is critical [15]. The absence of statistically significant differences across the software programs suggests that, within the contexts tested, all systems perform similarly, which is a reassuring outcome for practitioners. Nevertheless, further research incorporating larger sample sizes and additional factors, such as varying anatomical complexities and different implant scenarios, will be essential for comprehensively evaluating the performance of these software systems.

Limitations

While conducting the studies, we came across certain drawbacks which limit our study at this juncture of time. The smaller sample size of the study contributes to the limited variability and poses a question of the generalizability of this study. The use of goat mandibles as stand-in for human mandibles poses questions as to whether the anatomical differences can affect the findings of our study. Two observers participated in the study which leaves room for intra-observer and inter-observer bias. The scan was performed by a single CBCT scanning device to standardize the exposure parameters which limits the study from exploring the effects variable parameters can have on the image resolution and magnification and their role in linear measurements. We focused on only three 3D imaging software for comparison which restricts the scope of variations seen in other software with varying measuring tools. This study reasserts the fact that there is a need for further studies with larger sample sizes conducted by multiple examiners making use of more than three 3D viewing software to give a more multi-centric approach to the nature of this study.

## Conclusions

In our study, we compared three software keeping in mind their ability to make accurate linear measurements of an implant site using dry goat mandibles. It was concluded that all three software performed fairly well with CareStream and iRYSproducing results closer to the gold standard at lower error thresholds of 0.15 mm and 1.05 mm slice thicknesses and RadiAnt producing results closer to the gold standard at high error thresholds of 1.50 mm slice thickness. The results were consistent throughout the sample size, further proving that CBCT is an accurate measuring tool for implant planning second to the physical measurements.

While the current study provides valuable insights into the comparative accuracy of three competent 3D imaging software programs for alveolar bone measurements, it also highlights the need for ongoing research. Future studies could explore the impact of user experience, software updates, and varying imaging parameters on measurement accuracy. Additionally, implementing a broader range of thickness levels and anatomical variations could enhance our understanding of how these software solutions perform across different clinical scenarios. By continually assessing and refining these tools, clinicians can improve the precision of implant planning, ultimately enhancing patient outcomes.
